# Case Report: Successful treatment of pyoderma gangrenosum-like granulomatous liver disease without skin lesions using a TNF-alpha inhibitor

**DOI:** 10.3389/fimmu.2026.1811910

**Published:** 2026-06-15

**Authors:** Isabelle Panne, Matthias Matter, Jürg Vosbeck, Matthias von Rotz, Alexandra Steinemann, Marten Trendelenburg, Markus H. Heim, Peter Schirmacher, Andrea De Gottardi, Ansgar W. Lohse, Luigi M. Terracciano, Christine Bernsmeier

**Affiliations:** 1 University Centre for Gastrointestinal and Liver Diseases, University Hospital, Basel, Switzerland; 2 Department of Pathology, University Hospital, Basel, Switzerland; 3 Department of Infectious Diseases, University Hospital, Basel, Switzerland; 4 Department of Ophthalmology, University Hospital, Basel, Switzerland; 5 University Hospital Basel and Clinical Immunology, Department of Biomedicine, University of Basel, Basel, Switzerland; 6 Departments of Biomedicine and Clinical Research, University of Basel, Basel, Switzerland; 7 Department of Pathology, University Hospital Heidelberg, Heidelberg, Germany; 8 Gastroenterology and Hepatology, Luzerner Kantonsspital, Luzern, Switzerland; 9 Università della Svizzera Italiana, Faculty of Biomedical Sciences, Lugano, Switzerland; 10 Department of Medicine, University Medical Centre Hamburg-Eppendorf, Hamburg, Germany; 11 Department of Biomedical Sciences, Humanitas University, Pieve Emanuele, Milan, Italy; 12 IRCCS Humanitas Research Hospital, Rozzano, Milan, Italy

**Keywords:** extracutaneous manifestation, granulomatous liver disease, pyoderma gangrenosum, TNF – alpha inhibitor, treatment

## Abstract

Pyoderma gangrenosum is a rare autoimmune condition that is difficult to diagnose and primarily affects the skin. Extracutaneous manifestations, including hepatic involvement, occur only rarely. Here, we report a case of extracutaneous manifestation of pyoderma gangrenosum without adjunct skin lesions, emphasizing the diagnostic challenges, immunological considerations, and therapeutic implications. This case describes a 73-year-old man presenting with isolated hepatic and ocular involvement of pyoderma gangrenosum, without skin lesions and without an underlying chronic inflammatory disease. This case aims to expand the clinical understanding of the spectrum of pyoderma gangrenosum, reinforce its systemic nature, and contribute to improved recognition of extracutaneous disease involvement even without primary skin manifestation. The patient exhibited fever, weight loss of 3 kg within 3 weeks, elevated hepatocellular and cholestatic liver parameters (AST: 128 U/L, upper limit norm [ULN]: 34 U/L; ALT: 116 U/L, ULN: 59 U/L), and elevated CRP (147.5 mg/L, ULN: 10 mg/L). Imaging revealed disseminated nodular liver lesions. Histology demonstrated sterile abscesses and granulomatous inflammation. Ophthalmologic examination revealed conjunctival injections and granulomatous uveitis. No other organs were involved. Extensive testing excluded infectious or other immunological causes. After comprehensive screening for infectious, rheumatological, malignant, or hematological diseases, systemic inflammatory syndromes, and drug-induced liver injury, none of the suspected common diagnoses could be confirmed. In relation to the distinctive histological pattern resembling a few published case reports, we assumed pyoderma gangrenosum with hepatic and ocular involvement without skin lesions. We initiated immunosuppressive treatment with prednisone, followed by infliximab. This strategy successfully led to symptom resolution and complete clinical and biochemical remission. This case highlights the diagnostic challenges of granulomatous liver injury and pyoderma gangrenosum at unusual sites. Pyoderma gangrenosum is a rare condition, particularly when presenting with isolated hepatic and ocular involvement, and may be considered a differential diagnosis underlying granulomatous hepatic injury. Using a TNF-alpha inhibitor, we successfully achieved a complete remission.

## 1 Introduction

Pyoderma gangrenosum is a rare inflammatory neutrophilic dermatosis characterized by painful, rapidly progressive ulcerations with undermined violaceous borders and peripheral erythema (1–3). Although historically regarded as a primary cutaneous disorder, pyoderma gangrenosum is now increasingly understood as a systemic autoinflammatory disease driven by dysregulated innate immunity, aberrant neutrophil activation, and excessive proinflammatory cytokine signaling (1, 2). Pyoderma gangrenosum is frequently associated with immune-mediated comorbidities, most commonly inflammatory bowel disease, inflammatory arthritis, and hematologic malignancies, further supporting its systemic nature (1, 3).

While the skin remains the primary site of involvement, extracutaneous manifestations of pyoderma gangrenosum are rare but clinically significant. Often presenting as sterile neutrophilic infiltrates, the lung is the most frequently described extracutaneous site, followed by ocular involvement and other organ manifestations in the liver, spleen, bone, kidney, and gallbladder (3–8). Hepatic involvement appears to be particularly rare (9, 10). These manifestations underscore the systemic nature of pyoderma gangrenosum and highlight the need for heightened clinical awareness, especially as they frequently mimic infectious, malignant, or vasculitic conditions and may lead to delayed diagnosis, unnecessary antimicrobial therapy, or invasive procedures (5, 7).

Here, we report a case of extracutaneous manifestation of pyoderma gangrenosum, emphasizing the diagnostic challenges, immunological considerations, and therapeutic implications. This case aims to expand clinical awareness of the spectrum of pyoderma gangrenosum, reinforce its systemic nature, and contribute to improved recognition of extracutaneous disease involvement in neutrophilic dermatoses.

## 2 Case description

A 73-year-old man was admitted to our tertiary referral center with unclear hepatitis and multiple liver lesions of unknown etiology. He reported a history of several weeks of fever, night sweats, and weight loss. Three weeks before admission, he was treated with ciprofloxacin for 10 days because of a urinary infection. Although the urinary infection was resolved, the patient’s general condition did not improve, and he presented to his general physician with persisting fever and fatigue. Of note, the patient had a history of hepatitis B virus (HBV) infection with virologic response under tenofovir disoproxil fumarate (TDF) for years. Apart from arterial hypertension, prostate hyperplasia, depressive episodes, and hypogonadism, no relevant medical history was known. His medication included venlafaxine, valsartan, amlodipine, testosterone, tamsulosin, and TDF. The family history was negative for hepatic or autoimmune diseases. Originating from Italy, he had been living in Switzerland for decades. Computed tomography (CT) was initiated primarily to rule out malignancy. It revealed hepatomegaly with diffuse hypoechoic areas, and the patient was referred to our hepatology ward for further diagnostics and treatment.

At admission, he suffered mainly from fatigue and nausea and appeared slightly confused. There was no vomiting, and bowel movements were normal without any bleeding or diarrhea. Clinical examination was normal except for mild right upper quadrant abdominal discomfort on palpation and discrete conjunctival injections without pain or visual impairment. The patient had normal blood pressure, heart rate, and temperature. The skin condition was unremarkable; specifically, no pyoderma gangrenosum-like lesions were found.

Blood analyses revealed a mixed pattern of hepatitis with elevated transaminases and gamma-glutamyltransferase (GGT)/alkaline phosphatase (AP) (see also timeline). C-reactive protein (CRP) was markedly elevated (147 mg/L) without leucocytosis or neutrophilia. Further blood tests showed no evidence of metabolic, hereditary, or autoimmune liver diseases, although immunoglobulin G (IgG)1/4 and soluble IL-2 receptor were slightly elevated, and cryoglobulinemia was present. Moreover, the findings were not consistent with sarcoidosis, IgG4-related diseases, or vasculitis, which had been considered rare differential diagnoses during the diagnostic workup. Under ongoing treatment with TDF, the HBV viral load remained suppressed. Tumor markers, including alpha-fetoprotein, carbohydrate antigen 19-9, carcinoembryonic antigen, and prostate-specific antigen, were negative. There was no evidence of underlying lymphoma or other hematologic, malignant, or systemic inflammatory conditions after extensive investigations. Thus, a paraneoplastic inflammatory condition was ruled out. Endoscopy of the upper and lower GI tract and magnetic resonance imaging (MRI) enteroclysis did not reveal signs of inflammatory bowel disease. No history or recent exposure to new drugs was detected, making drug-induced liver injury improbable. To rule out a potential infectious inflammatory condition, extensive blood and liver tissue analyses, including metagenomic sequencing, were performed; however, they revealed no evidence of viral, bacterial, fungal, or parasite pathogens. These microbiological investigations encompassed all granulomatous liver disease-causing pathogens described in the literature, including mycobacterial diseases, fungal infections, bartonellosis, brucellosis, trichinellosis, toxocariasis, tularemia, leptospirosis, diverse parasitic infections such as schistosomiasis, toxoplasmosis, filariasis, fascioliasis, leishmaniasis, helminths, and amoebas. The following contrast-enhanced CT scans and MRI showed disseminated liver lesions. The liver showed normal perfusion of portal veins, hepatic veins, and hepatic arteries without arterial enhancement or washout patterns of the liver lesions. The intra- and extrahepatic bile ducts were normal, without caliber irregularities.

**Figure 1:**
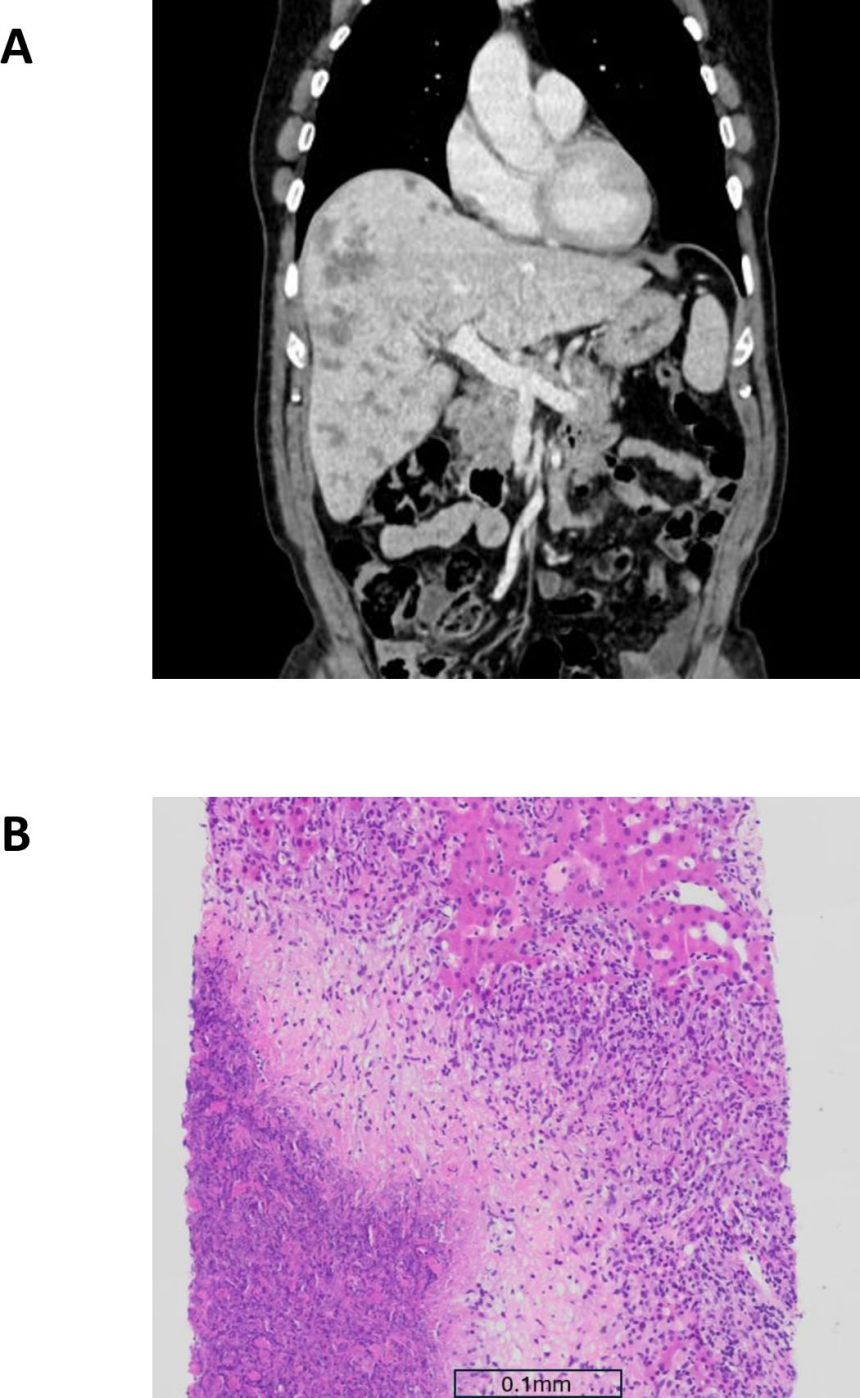
Hepatic manifestation of the suspected pyoderma gangrenosum. **(A)** CT imaging of the liver at admission showed multiple hypodense lesions in the whole liver, focused in the right liver lobe. **(B)** Histopathology (HE) showing multiple sterile, neutrophilic abscesses and necrosis with partly fibrotic granulomatous inflammation. Diffuse sinusoidal dilatation with partly necrotic vessels due to thrombotic occlusion.

**Figure 2:**
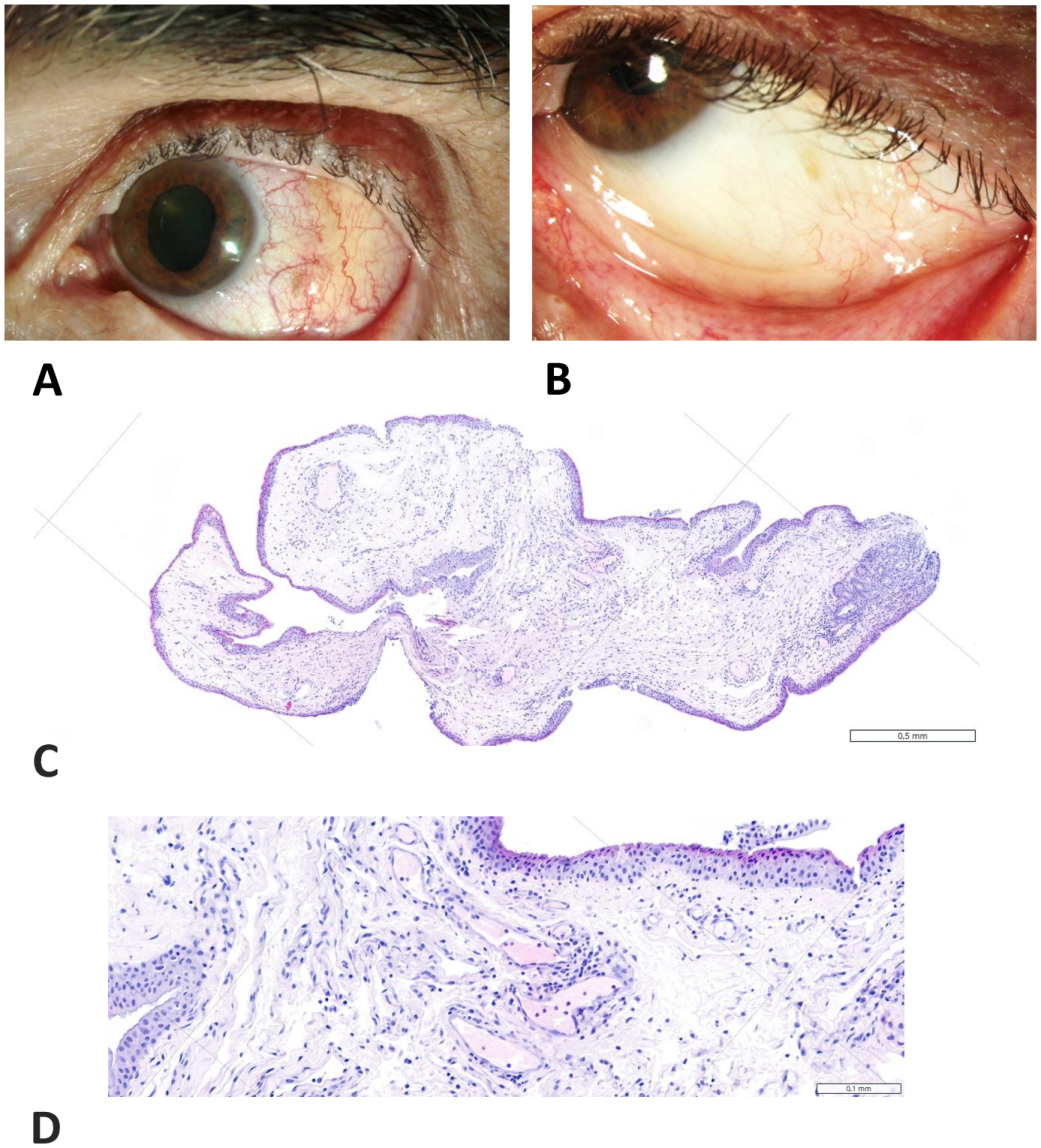
Ocular involvement. **(A)** Left eye at the time of diagnosis with conjuncival injections as clinical manifestation of the inflammation and **(B)** Right eye after 4 months of treatment. We see a normalized tissue with only slightly accentuatued vessels. **(C, D)** Histology of the left conjunctiva (HE) showing oedematous tissue with ectatic vessels containing thrombocyte–von Willebrand factor thrombi.

Histology of the liver showed disseminated sterile abscesses with areas of necrosis, fibrinous and chronic granulomatous inflammation, representing 50% of the parenchymal surface, as well as sinusoidal dilatation with thrombosis of larger vessels, suggesting microthrombosis leading to necrosis (**Figures 1**, **2**). In detail, histopathology revealed pan-acinar necrosis and histiocytosis, with an initial suspicion of a veno-occlusive disease; however, in the absence of hematologic malignancy, the findings were interpreted as central vein occlusion with perifocal reactive tissue. IgG4 and amyloid stainings were negative. Mild to moderate mononuclear infiltrate was seen in some portal fields with minimal interface activity. Pan-bacterial, pan-viral, and pan-fungal PCR results of the liver tissue were negative.

Given the histological pattern, other organs were investigated for possible granulomatous inflammation despite the lack of specific symptoms. Ophthalmologic slit-lamp examination revealed conjunctival injection with areas of scleritis and yellowish patchy changes of the conjunctiva, as well as simultaneous granulomatous uveitis anterior on both eyes. Histopathology of a conjunctival biopsy demonstrated a polypoid, tumor-free, edematous tissue with mild lymphoplasmacytic and neutrophilic infiltration, presence of mast cells, and ectatic vessels containing thrombocyte–von Willebrand factor thrombi, similar to the findings in liver tissue.

Discussing the etiology of this granulomatous liver disease with fever, weight loss, uveitis, and sterile, neutrophilic abscess-like histopathological findings in the liver and uvea, infectious causes, malignancy, including hematological malignancies, rheumatologic disease, and other sterile systemic inflammatory diseases such as VEXAS syndrome, vasculitis, and sarcoidosis were excluded. Inflammatory pseudotumor or a veno-occlusive disease were also considered possible differential diagnoses.

In relation to the literature and histopathological review by an international histopathological and hepatological expert panel from the VALDIG and GNOMES consortium, we finally postulated the diagnosis of a pyoderma gangrenosum with hepatic and ocular involvement, without skin lesions or underlying inflammatory bowel disease (IBD). Although the Delphi criteria designed for cutaneous ulcerative disease may not be completely applicable here, histopathology represented a major criterion to diagnose pyoderma gangrenosum (11). The rapid response to immunosuppression in our case with prednisone can be interpreted as another diagnostic criterion. Evidence of similar ulcerative patterns in two different tissues supported our suspicion of multilocular inflammatory involvement, serving as an additional minor criterion for the probable diagnosis of pyoderma gangrenosum, which we subsequently adopted as our working hypothesis.

In accordance, antibiotic treatment with piperacillin/tazobactam 4.5 g i.v. TID did not lead to improvement. Immunosuppressive therapy with 60 mg prednisone was therefore initiated, resulting in normalization of CRP and liver enzymes within 3 months. Steroid therapy was tapered once the liver parameters improved. Attempts to withdraw prednisone, however, induced a relapse of elevated liver parameters and CRP, necessitating reintroduction. Close monitoring (every 2 to 4 weeks) was carried out over a duration of 6 months, showing no relevant steroid-related complications or toxicity. The occurrence of recurrent urinary tract infections due to prostate hyperplasia may have been facilitated by immunosuppression. We subsequently changed to a steroid-sparing regimen with infliximab at a dosage of 5 mg/kg body weight every 4 weeks after ruling out contraindications (screening for tuberculosis, hepatitis C virus infection, no known malignancy, normal cardiac function). TDF for the known chronic HBV infection was continued, and permanent viral suppression was documented. Steroids were tapered over 12 weeks (details in **Supplementary Table 1**) and discontinued once the liver parameters normalized again. Under additional local prednisolone eye drops, ocular manifestations resolved (**Figure 2**). Due to the presence of thrombi in both the liver and the ocular tissue associated with necroses, anticoagulation with apixaban 5 mg BID was initiated.

Infliximab was well tolerated, and no relevant side effects or adverse events occurred during treatment over the 12-month follow-up period. Clinical, biochemical, radiologic, and ophthalmologic remission was achieved (for details, see **Supplementary Materials**). Infliximab was continued at 4-week intervals. As an adverse event, we report a prolonged urogenital infection episode over 3 months, attributed to prostate hyperplasia and complicated urinary tract infection. No other complications were observed.

## 3 Discussion

We highlight this case to emphasize the occurrence of pyoderma gangrenosum with isolated hepatic involvement and ocular manifestations, without underlying chronic inflammatory disease, as a rare condition. Diagnosis of pyoderma gangrenosum remains challenging due to the absence of pathognomonic laboratory or histopathological findings and the broad differential diagnosis, which may delay treatment and lead to complications. To address this issue, Delphi-based diagnostic criteria for ulcerative pyoderma gangrenosum have been proposed, incorporating histopathology, clinical features, exclusion of infection, pathergy, systemic associations, and therapeutic response to immunosuppression (3, 11). However, these criteria were primarily developed for classic cutaneous ulcerative disease and are less readily applicable in cases dominated by extracutaneous involvement.

From an immunological perspective, extracutaneous manifestations reinforce the concept of pyoderma gangrenosum as a systemic neutrophilic disease rather than a skin-restricted entity. Pathogenic mechanisms include exaggerated neutrophil recruitment, Th1/Th17-skewed immune responses, inflammasome activation, and overexpression of cytokines such as interleukin-1β, interleukin-17, interleukin-23, and tumor necrosis factor-α (TNF- α) (1, 2). Trauma-induced pathergy and genetic susceptibility may further amplify inflammatory cascades, contributing to disease dissemination beyond the skin (1).

The treatment of pyoderma gangrenosum is primarily based on immunosuppression and immunomodulation, reflecting its underlying autoinflammatory pathogenesis. Systemic corticosteroids and cyclosporine are considered first-line therapies for moderate-to-severe disease because of their rapid and broad anti-inflammatory effects and demonstrated efficacy in randomized controlled trials (1, 2, 12). These agents are often administered alone or in combination, with treatment choice guided by disease severity, comorbidities, and contraindications.

In patients with refractory, relapsing, or steroid-dependent inflammatory disease, biological therapies have emerged as effective options. Tumor necrosis factor-α inhibitors, particularly infliximab and adalimumab, have the strongest evidence base and are widely used, especially in pyoderma gangrenosum associated with inflammatory bowel disease or other systemic inflammatory conditions (1–3). Infliximab has demonstrated superiority over placebo in the only randomized, double-blind, placebo-controlled trial conducted for pyoderma gangrenosum. In this landmark study of 30 patients, infliximab at a dose of 5 mg/kg body weight produced a significantly higher response rate at 2 weeks (46% vs. 6%, *p* = 0.025) compared to placebo, with a 21% complete resolution rate at 6 weeks. When considering open-label treatment, 69% of patients demonstrated a beneficial clinical course (13). This represents the highest level of evidence available for any pyoderma gangrenosum therapy (14–16). Arguments supporting infliximab as a first-line agent include its rapid onset of action, high overall response rates, and comparable efficacy to traditional first-line agents such as steroids or cyclosporine, with fewer side effects. Additionally, infliximab offers distinct advantages in patients with concomitant inflammatory comorbidities, particularly IBD. Current expert consensus recognizes that biologics have become the first-line treatment of choice in patients with underlying inflammatory comorbidities (17–19). Newer data suggest that there is no significant difference in effectiveness among infliximab, adalimumab, and etanercept (15). The optimal duration of treatment remains unclear. Targeted therapies, including inhibitors of interleukin-1, interleukin-17, and interleukin-23, as well as complement C5a blockade and Janus kinase inhibitors, have shown promising results in case series and early clinical trials, particularly in refractory cases as second-line treatments (2, 12, 17, 19, 20). Moreover, mycophenolate mofetil is an effective steroid-sparing agent, achieving improvement in 93% of patients at a median dose of 2,000 mg daily, with better tolerability than cyclosporine (21).

An additional particularity of this case is the situation of an individual with chronic hepatitis B virus infection undergoing immunosuppressive therapy for another chronic liver disease. Immunosuppression may cause reactivation of hepatitis B. Close follow-up with regular monitoring of the HBV viral load every 3–6 months is recommended. Patients receiving established treatment for HBV infection should continue therapy during immunosuppressive treatment (22, 23).

## 4 Patient perspective

From the patient’s perspective, prolonged hospitalization and the extended time required to establish a reasonable diagnosis enabling a successful treatment caused significant stress and emotional strain. Persistent uncertainty about the diagnosis contributed to feelings of anxiety and lack of control throughout the course of the disease. Additionally, the patient suffered recurrent urogenital infections during immunosuppressive treatment. Upon initiation of anti-TNF therapy and tapering of steroids, his quality of life improved markedly. At present, the patient is in complete clinical remission. He reports no relevant side effects from the treatment and has resumed full participation in daily activities. The plan is to continue treatment with infliximab monotherapy.

## Data Availability

The original contributions presented in the study are included in the article/**Supplementary Material**. Further inquiries can be directed to the corresponding author.

## References

[1] Maverakis E Marzano AV Le ST Callen JP Brüggen MC Guenova E . Pyoderma gangrenosum. Nat Rev Dis Primer. (2020) 6:81. doi: 10.1038/s41572-020-0213-x 33033263

[2] Maronese CA Pimentel MA Li MM Genovese G Ortega-Loayza AG Marzano AV . Pyoderma gangrenosum: an updated literature review on established and emerging pharmacological treatments. Am J Clin Dermatol. (2022) 23:615–34. doi: 10.1007/s40257-022-00699-8 35606650 PMC9464730

[3] Zaino ML SChadt CR Callen JP . Pyoderma gangrenosum. Dermatol Clin. (2024) 42:157–70. doi: 10.1016/j.det.2023.08.003 38423678

[4] Miserocchi E Modorati G Foster CS Brancato R . Ocular and extracutaneous involvement in pyoderma gangrenosum. Ophthalmology. (2002) 109:1941–3. doi: 10.1016/S0161-6420(02)01165-X 12359619

[5] Reynolds C Schofer N Zengin E Lohse AW Faiss S Schmiedel S . Multiple abszesse nach südamerikakreuzfahrt. Internist. (2016) 57:284–8. doi: 10.1007/s00108-015-0004-8 26782091

[6] Vadillo M Jucgla A Podzamczer D Rufi G Domingo A . Pyoderma gangrenosum with liver, spleen and bone involvement in a patient with chronic myelomonocytic leukaemia. Br J Dermatol. (1999) 141:541–3. doi: 10.1046/j.1365-2133.1999.03055.x 10583064

[7] Borda LJ Wong LL Marzano AV Ortega-Loayza AG . Extracutaneous involvement of pyoderma gangrenosum. Arch Dermatol Res. (2019) 311:425–34. doi: 10.1007/s00403-019-01912-1 30923901

[8] Matsumura Y Nishiwaki F Morita N Kore-Eda S Miyachi Y . Pyoderma gangrenosum in a patient with myelodysplastic syndrome followed by possible extracutaneous manifestations in the gallbladder, liver, bone and lung. J Dermatol. (2011) 38:1102–5. doi: 10.1111/j.1346-8138.2010.01168.x 21434984

[9] Vadillo M Jucgla A Podzamczer D Rufi G Domingo A . Pyoderma gangrenosum with liver, spleen and btone involvement in a patient with chronic myelomonocytic leukaemia. Br J Dermatol. (1999) 141:541–3. doi: 10.1046/j.1365-2133.1999.03055.x 10583064

[10] Hanami Y Mori T Kikuchi N Yamamoto T . Association of pyoderma gangrenosum, erythema nodosum and aseptic liver abscess without significant underlying disease. Clin Exp Dermatol. (2019) 44:e16–7. doi: 10.1111/ced.13838 30460715

[11] Maverakis E Ma C Shinkai K Fiorentino D Callen JP Wollina U . Diagnostic criteria of ulcerative pyoderma gangrenosum: a delphi consensus of international experts. JAMA Dermatol. (2018) 154:461. doi: 10.1001/jamadermatol.2017.5980 29450466

[12] Alavi A French LE Davis MD Brassard A Kirsner RS . Pyoderma gangrenosum: an update on pathophysiology, diagnosis and treatment. Am J Clin Dermatol. (2017) 18:355–72. doi: 10.1007/s40257-017-0251-7 28224502

[13] Brooklyn TN Dunnill MGS Shetty A Bowden JJ Williams JDL Griffiths CEM . Infliximab for the treatment of pyoderma gangrenosum: a randomised, double blind, placebo controlled trial. Gut. (2006) 55:505–9. doi: 10.1136/gut.2005.074815 16188920 PMC1856164

[14] Eleftheriotis G Fragonikolaki M Karelaki C Syrigou E Georgiadis S Georgiadi K . Epidemiology, clinical data, and management of aseptic abscess syndrome: review of published cases outside France. Epidemiologia. (2025) 6:44. doi: 10.3390/epidemiologia6030044 40843703 PMC12371957

[15] Ben Abdallah H Fogh K Bech R . Pyoderma gangrenosum and tumour necrosis factor alpha inhibitors: a semi‐systematic review. Int Wound J. (2019) 16:511–21. doi: 10.1111/iwj.13067 30604927 PMC7949186

[16] Choe SI Shea M Oberg M Paz Munoz E Ortega-Loayza AG . Pyoderma gangrenosum with extracutaneous involvement: a systematic review. Clin Exp Dermatol. (2025) 50:1443–6. doi: 10.1093/ced/llaf104 40056453

[17] Moltrasio C Romagnuolo M Tavoletti G Maronese CA Marzano AV . Pyoderma gangrenosum: pathogenetic mechanisms and their implications for treatment. Semin Immunopathol. (2025) 47:38. doi: 10.1007/s00281-025-01064-7 41128863 PMC12549756

[18] Tan MG Tolkachjov SN . Treatment of pyoderma gangrenosum. Dermatol Clin. (2024) 42:183–92. doi: 10.1016/j.det.2023.12.002 38423680

[19] Partridge ACR Bai JW Rosen CF Walsh SR Gulliver WP Fleming P . Effectiveness of systemic treatments for pyoderma gangrenosum: a systematic review of observational studies and clinical trials. Br J Dermatol. (2018) 179:290–5. doi: 10.1111/bjd.16485 29478243

[20] Dissemond J Marzano AV Hampton PJ Ortega-Loayza AG . Pyoderma gangrenosum: treatment options. Drugs. (2023) 83:1255–67. doi: 10.1007/s40265-023-01931-3 37610614 PMC10511384

[21] Hrin ML Bashyam AM Huang WW Feldman SR . Mycophenolate mofetil as adjunctive therapy to corticosteroids for the treatment of pyoderma gangrenosum: a case series and literature review. Int J Dermatol. (2021) 60:e486–92. doi: 10.1111/ijd.15539 33739458

[22] Ali FS Nguyen MH Hernaez R Huang DQ Wilder J Piscoya A . AGA clinical practice guideline on the prevention and treatment of hepatitis B virus reactivation in at-risk individuals. Gastroenterology. (2025) 168:267–84. doi: 10.1053/j.gastro.2024.11.008 39863345

[23] Cornberg M Sandmann L Jaroszewicz J Kennedy P Lampertico P Lemoine M . EASL clinical practice guidelines on the management of hepatitis B virus infection. J Hepatol. (2025) 83(2):502–83. doi: 10.1016/j.jhep.2025.03.018 40348683

