# Supplementary material for: Case Report: Successful treatment of pyoderma gangrenosum-like granulomatous liver disease without skin lesions using a TNF-alpha inhibitor
Source: Front Immunol. 2026 Jun 15;17:1811910. doi: 10.3389/fimmu.2026.1811910 (PMC13311106; doi:10.3389/fimmu.2026.1811910)
Supplement: Supplementary file 1 [file Presentation1.pptx]

## Slide 1
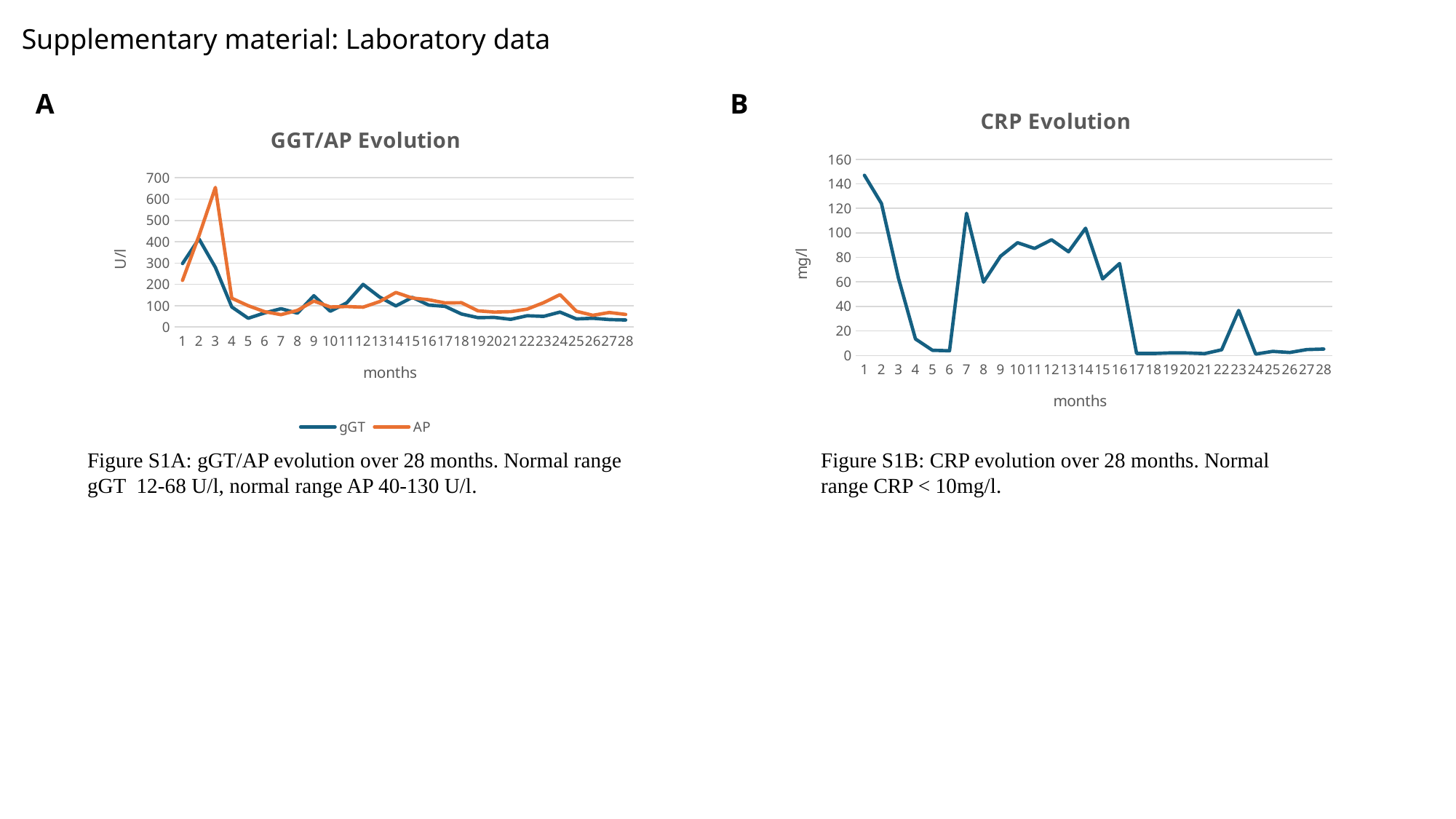

Supplementary material: Laboratory data
A
B
### Chart: CRP Evolution
| Category | CRP (mg/l) |
|---|---|
| 1 | 147.0 |
| 2 | 124.0 |
| 3 | 63.0 |
| 4 | 13.4 |
| 5 | 4.2 |
| 6 | 3.8 |
| 7 | 116.0 |
| 8 | 59.8 |
| 9 | 81.0 |
| 10 | 92.0 |
| 11 | 87.3 |
| 12 | 94.4 |
| 13 | 84.6 |
| 14 | 103.8 |
| 15 | 62.4 |
| 16 | 75.0 |
| 17 | 1.6 |
| 18 | 1.6 |
| 19 | 2.1 |
| 20 | 2.0 |
| 21 | 1.5 |
| 22 | 4.6 |
| 23 | 36.7 |
| 24 | 1.1 |
| 25 | 3.3 |
| 26 | 2.4 |
| 27 | 4.8 |
| 28 | 5.2 |
### Chart: GGT/AP Evolution
| Category | gGT | AP |
|---|---|---|
| 1 | 298.0 | 219.0 |
| 2 | 414.0 | 428.0 |
| 3 | 280.0 | 655.0 |
| 4 | 94.0 | 135.0 |
| 5 | 41.0 | 100.0 |
| 6 | 66.0 | 72.0 |
| 7 | 86.0 | 58.0 |
| 8 | 65.0 | 78.0 |
| 9 | 147.0 | 122.0 |
| 10 | 74.0 | 94.0 |
| 11 | 113.0 | 96.0 |
| 12 | 200.0 | 93.0 |
| 13 | 141.0 | 119.0 |
| 14 | 99.0 | 162.0 |
| 15 | 139.0 | 136.0 |
| 16 | 103.0 | 128.0 |
| 17 | 97.0 | 113.0 |
| 18 | 61.0 | 114.0 |
| 19 | 44.0 | 76.0 |
| 20 | 45.0 | 70.0 |
| 21 | 36.0 | 72.0 |
| 22 | 53.0 | 84.0 |
| 23 | 50.0 | 114.0 |
| 24 | 70.0 | 152.0 |
| 25 | 38.0 | 74.0 |
| 26 | 41.0 | 55.0 |
| 27 | 35.0 | 68.0 |
| 28 | 33.0 | 59.0 |Figure S1A: gGT/AP evolution over 28 months. Normal range gGT 12-68 U/l, normal range AP 40-130 U/l.
Figure S1B: CRP evolution over 28 months. Normal range CRP < 10mg/l.
