# Supplementary material for: Case Report: Successful treatment of pyoderma gangrenosum-like granulomatous liver disease without skin lesions using a TNF-alpha inhibitor
Source: Front Immunol. 2026 Jun 15;17:1811910. doi: 10.3389/fimmu.2026.1811910 (PMC13311106; doi:10.3389/fimmu.2026.1811910)
Supplement: Supplementary file 2 [file Presentation2.pptx]

## Slide 1
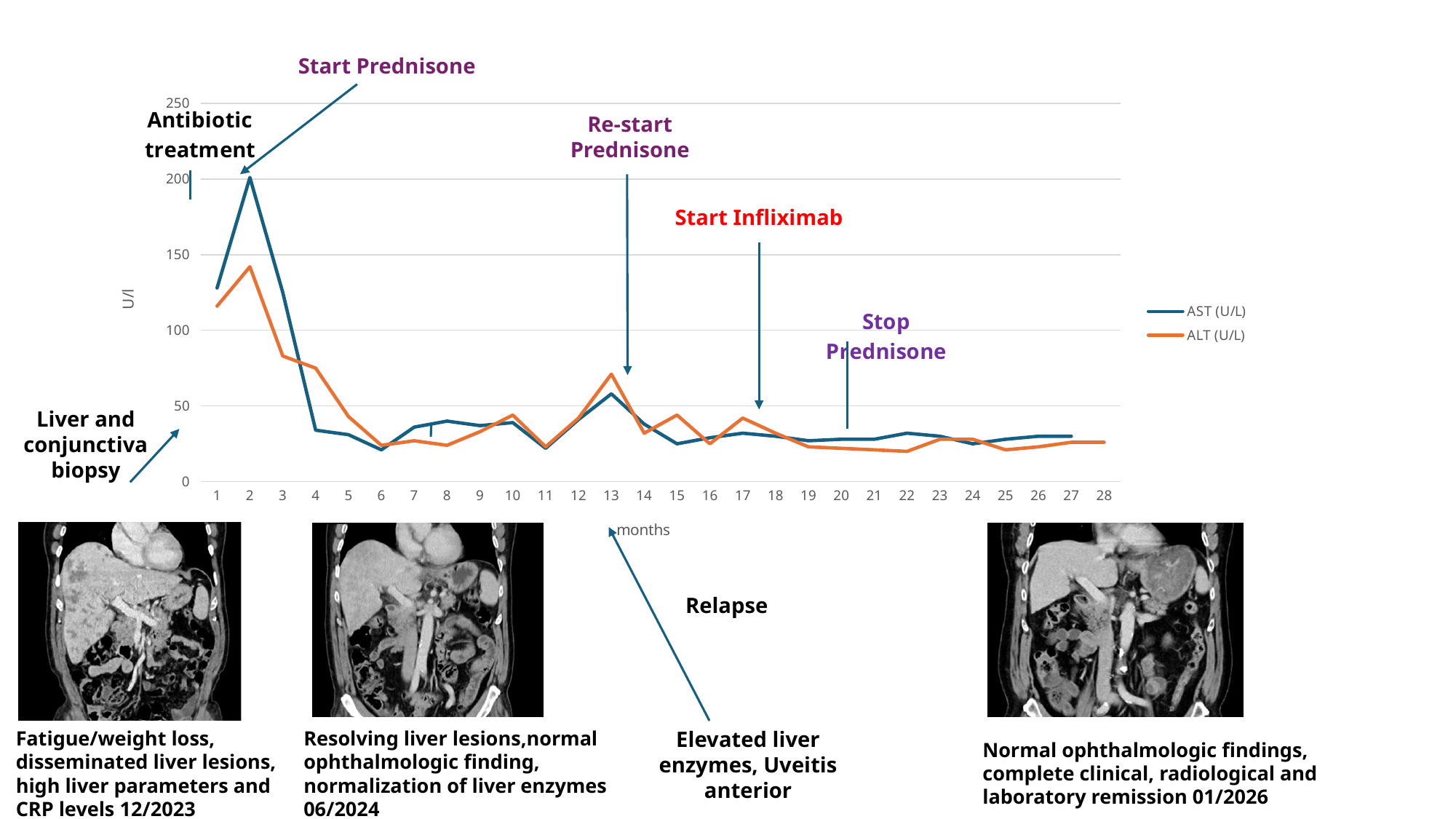

Start Prednisone
### Chart
| Category | AST (U/L) | ALT (U/L) |
|---|---|---|
| 1 | 128.0 | 116.0 |
| 2 | 201.0 | 142.0 |
| 3 | 125.0 | 83.0 |
| 4 | 34.0 | 75.0 |
| 5 | 31.0 | 43.0 |
| 6 | 21.0 | 24.0 |
| 7 | 36.0 | 27.0 |
| 8 | 40.0 | 24.0 |
| 9 | 37.0 | 33.0 |
| 10 | 39.0 | 44.0 |
| 11 | 22.0 | 23.0 |
| 12 | 41.0 | 42.0 |
| 13 | 58.0 | 71.0 |
| 14 | 38.0 | 32.0 |
| 15 | 25.0 | 44.0 |
| 16 | 29.0 | 25.0 |
| 17 | 32.0 | 42.0 |
| 18 | 30.0 | 32.0 |
| 19 | 27.0 | 23.0 |
| 20 | 28.0 | 22.0 |
| 21 | 28.0 | 21.0 |
| 22 | 32.0 | 20.0 |
| 23 | 30.0 | 28.0 |
| 24 | 25.0 | 28.0 |
| 25 | 28.0 | 21.0 |
| 26 | 30.0 | 23.0 |
| 27 | 30.0 | 26.0 |
| 28 | None | 26.0 |Re-start Prednisone
Start Infliximab
Liver and conjunctiva biopsy
Relapse
Resolving liver lesions,normal ophthalmologic finding, normalization of liver enzymes 06/2024
Elevated liver enzymes, Uveitis anterior
Fatigue/weight loss, disseminated liver lesions, high liver parameters and CRP levels 12/2023
Normal ophthalmologic findings, complete clinical, radiological and laboratory remission 01/2026
