# Supplementary material for: Case Report: Successful treatment of pyoderma gangrenosum-like granulomatous liver disease without skin lesions using a TNF-alpha inhibitor
Source: Front Immunol. 2026 Jun 15;17:1811910. doi: 10.3389/fimmu.2026.1811910 (PMC13311106; doi:10.3389/fimmu.2026.1811910)
Supplement: Supplementary file 3 [file Table1.docx]

Supplementary material:

Table 1: Prednisone tapering regimen

| **Duration** | **Daily Dosage (Prednisone)** |
| --- | --- |
| **Day 1-3** | 70 mg |
| **Week 1–2** | 30 mg |
| **Week 3–4** | 20 mg |
| **Week 5–7** | 10 mg |
| **Week 8-9** | 5 mg |
| **Week 10-12** | 2,5 mg |
